# The research progress on radiation resistance of cervical cancer

**DOI:** 10.3389/fonc.2024.1380448

**Published:** 2024-04-08

**Authors:** Meili Liang, Liying Sheng, Yumin Ke, Zhuna Wu

**Affiliations:** Department of Gynecology and Obstetrics, The Second Affiliated Hospital of Fujian Medical University, Quanzhou, Fujian, China

**Keywords:** cervical cancer, radiation resistance, tumor microenvironment, cancer stem cells, epigenetic mechanisms

## Abstract

Cervical carcinoma is the most prevalent gynecology malignant tumor and ranks as the fourth most common cancer worldwide, thus posing a significant threat to the lives and health of women. Advanced and early-stage cervical carcinoma patients with high-risk factors require adjuvant treatment following surgery, with radiotherapy being the primary approach. However, the tolerance of cervical cancer to radiotherapy has become a major obstacle in its treatment. Recent studies have demonstrated that radiation resistance in cervical cancer is closely associated with DNA damage repair pathways, the tumor microenvironment, tumor stem cells, hypoxia, cell cycle arrest, and epigenetic mechanisms, among other factors. The development of tumor radiation resistance involves complex interactions between multiple genes, pathways, and mechanisms, wherein each factor interacts through one or more signaling pathways. This paper provides an overview of research progress on an understanding of the mechanism underlying radiation resistance in cervical cancer.

## 1 Introduction

**Figure 1:**
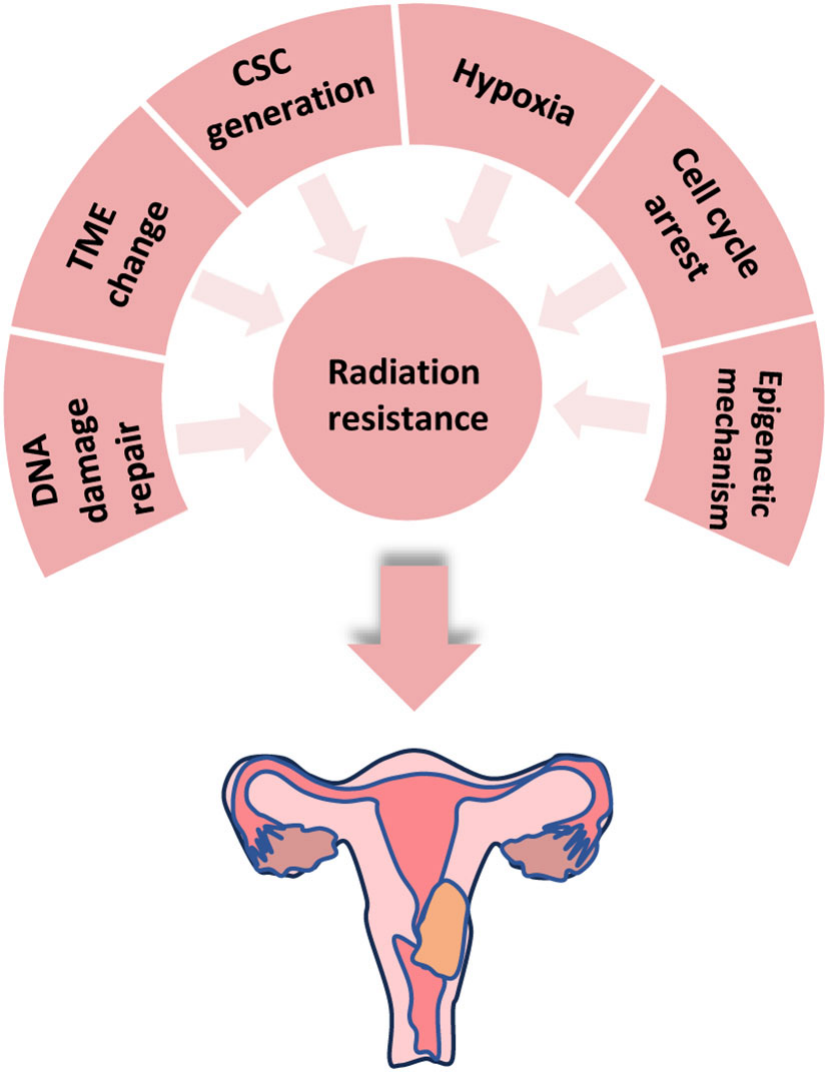
The mechanisms of radiotherapy tolerance in cervical cancer.

Cervical cancer not only ranks as the fourth most prevalent cancer worldwide but also remains one of the leading reasons for cancer-related mortality among women. An estimated 604,000 new cases of cervical cancer and 342,000 deaths were reported worldwide according to WHO statistics, in 2020 (1). The risk factors for cervical cancer include those associated with human papillomavirus (HPV) infection risk, as well as non-HPV-related factors such as HIV and *Chlamydia trachomatis* infections, smoking history, fertility issues, and long-term use of oral contraceptives (2). Radiotherapy (RT), which is a technique based on ionizing radiation, stands out as the most effective cytotoxic treatment option (3). RT plays a pivotal role in managing various cancers with approximately half of all cancer patients receiving this form of therapy (4). It can be employed for curative purposes or used postoperatively to eliminate residual disease or provide palliative care. In cases where surgical intervention is not feasible during the early stages of the disease, RT serves as a standalone treatment strategy (5). For cervical cancer management specifically, RT possesses considerable significance, particularly for locally advanced cases. Stage III-IVA cervical cancers exhibit high recurrence rates and poor survival outcomes. The five-year survival rates range between 39.7% and 41.5% for stage III disease and only reach 22% for stage IVA disease. Stage IIIB is typically treated solely with radiotherapy alone, thus resulting in a five-year survival rate of 46% (6). Concurrent chemoradiotherapy has been shown to improve the five-year survival rate by approximately 6%, thus yielding an overall five-year survival rate of approximately 70%, compared to radiotherapy alone among patients with locally advanced cervical cancer (2). However, tumor-specific radioresistance is the primary cause of radiotherapy failure, thus leading to local recurrence and distant metastasis. Tolerance is a commonly observed phenomenon during cervical cancer radiotherapy. The development of tumor radiation resistance involves a complex process encompassing multiple genes, pathways, and mechanisms. This article provides an overview of advancements in an understanding of DNA damage repair, the tumor microenvironment, tumor stem cells, hypoxia, cell cycle arrest, and epigenetic mechanisms contributing to the mechanism of radiation resistance in cervical cancer (**Figure 1**).

## 2 DNA damage repair

A hallmark of ionizing radiation is its ability to induce DNA damage, which directly contributes to carcinoma cell death. The critical mechanisms of DNA damage include breaks in the sugar-phosphate bond region (single-strand breaks, or SSBs), disruptions of adjacent or nearly adjacent sugar-phosphate binding sites (double-strand breaks, or DSBs), linkages between DNA or DNA-proteins, organic base degradation, depletion of purine or pyrimidine bases, and rupture of hydrogen bonds that result in permanent distortion of the DNA architecture (7), which are among the most lethal damage mechanisms(with DSBs being particularly detrimental). In mammalian cells, homologous recombination (HR) and DNA nonhomologous end joining (NHEJ) are the two primary DSB repair pathways. NHEJ predominates in DSB repair following radiation exposure, whereas HR plays a secondary role in DSB repair induced by radiation (5, 8).

### 2.1 Proto-oncogene

#### 2.1.1 MTDH gene

**Figure 2:**
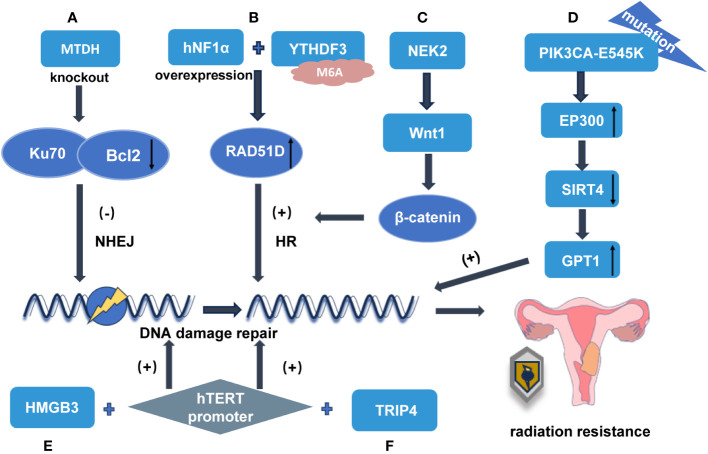
The mechanisms involved in DNA damage repair that cause radiation tolerance in cervical cancer **(A)** MTDH gene knockout decreased the expression of Ku70 and Bcl2 and enhanced the radiosensitivity of NHEJ. **(B)** After the overexpression of hNF1α bound to YTHDF3, YTHDF3 enhanced the radiation resistance of cervical cancer by increasing the expression of RAD51D in an m6A-dependent manner and thereby involved in DNA damage repair through the HR pathway. **(C)** NEK2 activates WNT/β-catenin signaling pathway by up-regulating WNT1 to enhance HR repair and promote radiation resistance. **(D)** PIK3CA-E545K negatively regulates SIRT4 expression by regulating the downstream effector P300 level, while SIRT4 inhibition leads to up-regulation of GPT1 expression, which enhances glutamine uptake and utilization, and ultimately promotes DNA damage repair. **(E)** The transcription factor HMGB3 binds to the hTERT promoter region and upregulates the expression of hTERT at the transcriptional level, which activates the DNA damage repair signaling pathway and promotes the radiation resistance of cervical cancer. **(F)** TRIP4 promotes radiation resistance of cervical cancer by targeting hTERT signal transduction and activating DNA damage repair signaling pathway by regulating its binding to the hTERT promoter.

The proteins involved in NHEJ include Ku70, Ku80, and DNA-PKcs, which together constitute the DNA-dependent protein kinase complex (DNA-PK) (9, 10). A previous study has shown that the knockout of the MTDH gene reduces the expression of Ku70 and Bcl2, which affects DSB repair by damaging NHEJ and improves radiation sensitivity (11) (**Figure 2A**).

#### 2.1.2 HNF1α gene

Hnf1α, which is a crucial member of the hepatocyte nuclear factor 1 (HNF1) transcription factor family, functions as a proto-oncogene in cervical cancer and actively participates in the proliferation and EMT of cervical cancer cells. Its overexpression significantly enhances the radiation resistance of cervical cancer cells both *in vitro* and *in vivo (* 12). N6-methyladenosine (M6A), which is the most significant RNA modification, plays a pivotal role in regulating RNA metabolism and is closely associated with tumor initiation and progression (13). RAD51D, which is an integral component of the RAD51-like protein complex, primarily operates within the homologous recombination repair pathway that is responsible for mending double-stranded DNA breaks induced by DNA-damaging agents during DNA replication (14). YTHDF3 facilitates the translation efficiency of its target mRNAs through an M6A methylation-dependent mechanism (15). The translation process mediated by Ythdf3 is hindered when MRGs such as SPI1 and PHF12 undergo reduced m6A modification, thereby promoting cancer progression (16).

HNF1α expression is significantly upregulated in radioresistant cervical cancer cells. Although HNF1α positively regulates RAD51 homolog 4 (RAD51D) at the protein level, there appears to be no direct interaction between these factors at the gene level. YTHDF3-mediated upregulation of m6A modifications promotes fatty acid metabolism and promotes lymphatic metastasis of cervical cancer by activating the LRP6-YAP-VEGF-C axis (17). Upon binding with overexpressed hNF1α, YTHDF3 accelerates RAD51D translation in an M6A-dependent manner to effectively regulate its expression levels. This regulatory mechanism involving YTHDF3-mediated HR pathway-based DNA damage repair contributes to the modulation of radiation resistance in cervical cancer (**Figure 2B**). The blockage of this pathway may offer therapeutic benefits for enhancing radiation tolerance by specifically targeting cervical cancer (18).

#### 2.1.3 Proto-oncogene WNT1 participates in the NEK2-WNT1-β-catenin signaling pathway

NEK kinases perform vital functions in governing cell cycle progression, organizing centrosomes, modulating RNA splicing, coordinating inflammatory responses, and regulating DNA damage repair mechanisms. NEK2 is frequently upregulated in various cancer types and contributes to tumor progression, metastasis, and resistance to therapeutic agents (19). For instance, NEK2 phosphorylates P53 at the Ser315 residue, thus leading to its destabilization. Consequently, this inhibits P53-mediated apoptosis and promotes tumorigenesis (20) The Wnt/β-Catenin pathway is a highly conserved signaling cascade that stringently regulates multiple cellular processes, including the initiation, progression, and invasion of cervical cancer, as well as its resistance to treatment (21). Tie Xu et al. demonstrated that the silencing of NEK2 significantly reduces the expression of Wnt1, which is a key downstream effector involved in transcriptional and translational regulation, thereby attenuating the Wnt/β-catenin signaling pathway. This effect resulted in impaired cell growth and proliferation along with increased radiosensitivity. By upregulating WNT1 expression levels, NEK2 activates the WNT/β-catenin signaling pathway, which promotes cervical cancer development while also conferring radiation resistance (19). The genetic knockout of the NEK2 gene accelerates DNA damage accumulation by impairing DNA repair mechanisms, thus ultimately enhancing radiosensitivity in cervical cancer cells. The results of Tie Xu demonstrated revealed that the silencing of NEK2 led to a significant reduction in radiation-induced Rad51 foci formation (19), thus indicating its close association with DNA damage repair processes and radiation resistance (**Figure 2C**). Fortunately, studies have identified XAV939 as an inhibitor targeting the Wnt signaling pathway, which effectively blocks β-catenin-dependent transcriptional activity, thus specifically improving radiosensitivity in HeLa cells (22).

### 2.2 Antioncogene

#### 2.2.1 The PIK3CA-E545K/SIRT4/GPT1 signaling pathway is involved in the antioncogene PIK3CA

Approximately 40% of cervical cancer cases exhibit mutations or amplifications in PIK3CA, which is the most prevalent genetic aberration observed in this type of cancer. Prior studies have demonstrated that the presence of the PIK3CA-E545K mutation confers radiation resistance in cervical cancer (23), thus indicating an unfavorable prognosis for patients with activated PIK3 signaling.

The mechanism of SIRT4 as a tumor suppressor can be essentially separated into two aspects. First, SIRT4 plays a crucial role in mitigating glutamine metabolism in mitochondria and controlling cell cycle progression and genome fidelity in response to DNA damage. The absence of SIRT4 results in heightened proliferation that is reliant on glutamine and exacerbated stress-induced genomic instability, leading to an oncogenic phenotype (24). Second, SIRT4 responds to the atypical activation of mTORC1 and regulates GDH activity, thereby facilitating the uptake and utilization of glutamine (25). A study conducted by W. Jiang et al. demonstrated that the PIK3CA-E545K mutation negatively regulates SIRT4 expression by modulating the levels of the downstream effector P300 (26). SIRT4 inhibition leads to the upregulation of GPT1 expression and enhanced glutamine uptake and utilization, which ultimately promotes DNA damage repair and affects radiosensitivity (**Figure 2D**). This was the first study to demonstrate that the PI3K activation regulates SIRT4 independent of the mTORC1 in cervical cancer. This result suggests that the synergistic effect of PI3K pathway inhibitor BYL719 combined with radiotherapy in the treatment of cervical cancer is better than that of mTOR inhibitor EVE, which is a significant finding for improving the radiosensitivity of cervical cancer (26). However, the negative regulatory effect of P300 on SIRT4 and the mechanism by which SIRT4 regulates GPT1 still need to be further studied.

#### 2.2.2 Human telomerase reverse transcriptase-related pathways

Telomeres are specialized DNA/protein structures that safeguard chromosomes against fusion and loss of coding sequences, thereby maintaining chromosomal stability (27). However, excessive telomerase activity is closely associated with tumorigenesis, metastasis, and the cancer stem cell phenotype (28). Human telomerase reverse transcriptase (HTERT) serves as the catalytic subunit of telomerase, thus preserving telomere integrity and participating in the DNA damage repair response induced by radiation. Moreover, it aids cells in overcoming apoptosis and cell cycle arrest, thus rendering carcinoma cells more resistant to chemotherapy and radiotherapy (29, 30). The high-mobility group box (HMGB) protein, which function as a DNA chaperone, is an omnipresent DNA-binding protein that is associated with chromatin in mammals. It plays a crucial role in replication, transcription, recombination, and repair processes (31). HMGB1 activates the ERK/MAPK, NF-κB, and Akt signaling pathways that lead to cancer cell reprogramming, by binding to the receptor for advanced glycation end products (RAGE). HMGB1 can promote the immune escape of cervical cancer by activating Tregs or facilitating Th2 polarization, so as to accelerate the metastasis of cervical cancer (32). Furthermore, the upregulation of HMGB1 was found to activate the DNA damage response by promoting chromatin modification and increasing CHK1 phosphorylation. This contributes to squamous cell carcinoma resistance towards to radiotherapy (33). This study demonstrated that the knockout of the HMGB1 gene leads to telomere dysfunction and a reduction in telomerase activity, whereas HMGB1 overexpression enhances the activity of telomerase (34). Both HMGB1 and HMGB2 possess the ability to bind to DNA without sequence specificity, thus facilitating the formation of nuclear protein complexes. Additionally, they can directly interact with DNA-binding proteins, thus subsequently influencing transcriptional processes. Research has indicated that HMGB3 promotes neoplasm development and sustains dedifferentiation in various cancers, including esophageal cancer, bladder cancer gastric cancer, breast cancer, non-small cell lung cancer, and hematologic diseases (35, 36). The inhibition of HMGB3 in ovarian carcinoma cells that are resistant to cisplatin can result in the downregulation of ATR and CHK1 transcription, as well as the attenuation of the ATR/CHK1/p-CHK1 DNA damage signaling pathway (35).

Recent findings from Li’s research have demonstrated that HMGB3 acts as a transcription factor, not only by enhancing the phosphorylation levels of p85, AKT, p110α, and p110β but also by reducing the phosphorylation level of pTEN. Moreover, HMGB3 combines with the promoter region of hTERT and stimulates its expression at the transcriptional level, thereby activating the DNA damage repair signaling pathway. Consequently, this contributes to cervical cancer resistance to radiation therapy, thus ultimately resulting in a poor prognosis (**Figure 2E**). Therefore, the targeting of the HMGB3/hTERT signaling axis may represent a vital strategy for enhancing radiotherapy tolerance in cervical cancer (37).

The subunit TRIP4 is a constituent of the transcription coactivator ASC-1, which performs a vital function in linking transcription factors and modifying chromatin structure. Che et al. found that TRIP4 exhibits elevated expression levels in cervical cancer cells. The knockout of TRIP4 significantly impedes the proliferation and EMT of cervical cancer cells, which is concurrent with the deactivation of the PI3K/AKT and MAPK/ERK signaling pathways. Moreover, TRIP4 has been found to regulate hTERT signaling by modulating its binding to the hTERT promoter region. Additionally, the knockdown of TRIP4 enhances cellular susceptibility to radiation (**Figure 2F**), thus leading to the downregulation of RAD51 and p-H2AX expression levels. Furthermore, *in vivo* studies have demonstrated that the knockdown of TRIP4 effectively inhibits cervical cancer growth and progression in a xenograft tumor model while concurrently reducing hTERT expression (38). The study by Lipinska et al. also demonstrated that downregulation of hTERT inhibits the PI3K/AKT signaling pathway in cervical cancer (29). However, the results of Lee et al. suggest that activated AKT not only stimulates c-Myc(thus resulting in cytoplasmic retention of BRCA-1 for negative regulation of hTERT activation and telomerase)but also, mediates phosphorylation events on hTERT to enhance telomerase activity (39). Therefore, the targeting of TRIP4 in conjunction with hTERT and the PI3K/AKT pathway can serve as potential therapeutic strategies for overcoming radiotherapy resistance in cervical cancer.

## 3 Tumor microenvironment

The tumor microenvironment (TME) is a specialized niche that fosters cancer progression (40). It is composed of endothelial cells, tumor-associated macrophages (TAMs), cancer-associated fibroblasts (CAFs), and other immune cells (41). Immune cell populations within the TME encompass M2 phenotype TAMs, N2 phenotype tumor-associated neutrophils, natural killer cells, mast cells, and myeloid-derived suppressor cells. A variety of proangiogenic factors, including cytokines, chemokines, and enzymes, are produced either indirectly or directly (42, 43).

### 3.1 Tumor-associated macrophages

**Figure 3:**
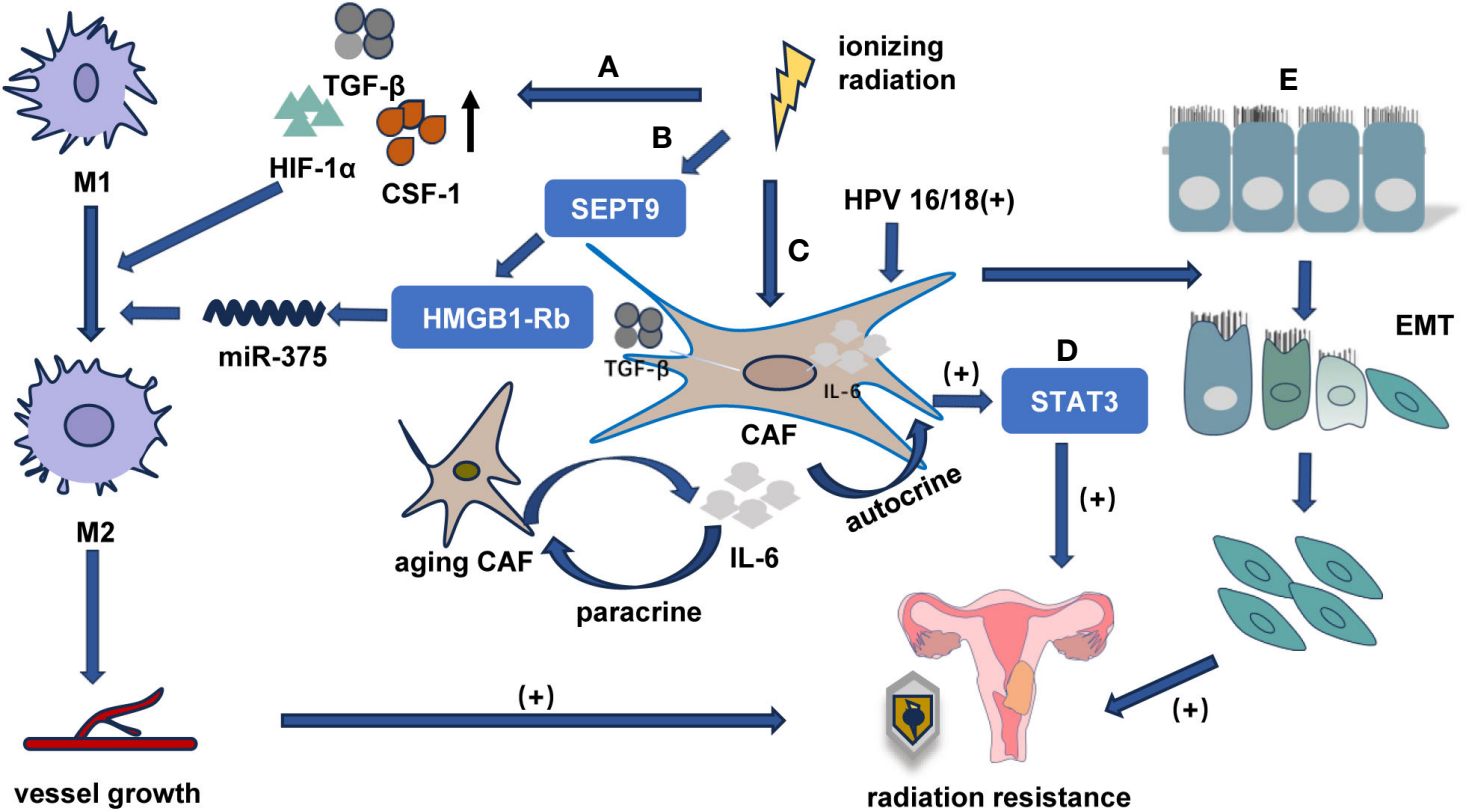
The relationship between TME and radiotherapy tolerance in cervical cancer **(A)** Radiation-induced activation of TGF-β, CSF-1, and HIF-1α in the TME promotes M1 macrophage differentiation into M2 phenotype, promoting abnormal angiogenesis, and enhancing radiotherapy resistance. **(B)** HPV16/18 infection activates the IL-6/STAT3 pathway through E6 protein, increasing IL-6 secretion and inducing senescence in CAFs through paracrine signaling. **(C)** Senescent CAFs secrete additional IL-6 that activates STAT3 in fibroblasts through an autocrine mechanism, enhancing cervical cancer resistance to radiotherapy. **(D)** Irradiated fibroblasts may enhance cancer cell invasiveness and promote EMT, thereby increasing radiation resistance. **(E)** SEPT9 targets the HMGB1-Rb pathway, facilitating M1 macrophage differentiation into M2 phenotype, promoting abnormal angiogenesis, and enhancing radiotherapy resistance.

TAMs can be classified into two distinct subtypes: classically activated macrophages (M1) and alternatively activated macrophages (M2). M1 macrophages, which exhibit a proinflammatory phenotype, express iNOS. In contrast, M2 macrophages secrete immunosuppressive cytokines, express Arg1 and CD206, and release IL-4 and IL-10 (44, 45). It has been noted that M1 macrophages participate in the normalization of tumor vasculature, whereas M2 macrophages contribute to the formation of abnormal blood vessels in tumors. Radiation exposure triggers the activation of TGF-β, CSF-1, and HIF-1α in the microenvironment, thus leading to differentiation from M1 to M2 (46) (**Figure 3A**).

TAMs, especially macrophages with M2 phenotype, reduce the survival rate of locally advanced cervical cancer patients by promoting the formation of new lymphatic vessels and blood vessels during cervical cancer metastasis (47). The high expression of M2 macrophage markers associated with tumor progression indicates an unfavorable prognosis. Lippens et al. demonstrated that patients with cervical cancer and HNSCC who exhibited elevated levels of the M2 marker CD163 had a poor prognosis following radiotherapy. Furthermore, Meng reported that the administration of macrophage-depleted liposomal krodrolic acid (either systemically or locally before radiotherapy) enhanced the efficacy of radiation treatment (44). Additionally, the study by Jiao et al. revealed for the first time that SEPT9 facilitates cell migration, invasion, and tumorigenesis and adjusts radiotherapy sensitivity by targeting the HMGB1-Rb pathway and modulating macrophage differentiation through miR-375 (**Figure 3B**). Therefore, the hypermethylation of the SEPT9 gene exhibits hypersensitivity and high specificity in diagnosing cervical carcinoma (48).

### 3.2 Tumor-associated fibroblasts

The association between CAFs and chemoresistance and radiation resistance has been previously established (49, 50). Ren et al. found that HPV16/18 infection in cervical cancer cells activates the IL-6/STAT3 pathway through the E6 protein, thus leading to the secretion of IL-6 into the extracellular matrix. This paracrine pathway induces senescence in interstitial fibroblasts. Conversely, senescent fibroblasts secrete elevated levels of inflammatory cytokines (including IL-6), which further activate STAT3 in an autocrine manner and regulate the tumor microenvironment, thus resulting in resistance and recurrence of cervical cancer after radiotherapy (51) (**Figures 3C, D**). Therefore, the HPV16/18E6 protein activates IL-6/STAT3 signaling to induce senescence in cancer-associated fibroblasts, thus making it a novel target for overcoming radiotherapy resistance. Irradiated fibroblasts possess the capacity to enhance cancer cell invasiveness and trigger epithelial-to-mesenchymal transition (**Figure 3E**). Subsequent to radiotherapy, tumor cells display an increased expression of transforming growth factor-β due to fibroblast activation. The subsequent upregulation of transforming growth factor-β signaling activates the TME and promotes tumor progression (52).

## 4 Cancer stem cells

Cancer stem cells (CSCs), which represent a minor subset of tumor cells defined by their self-renewal capacity, have been demonstrated to possess resistance to radiation at several sites (53, 54). Moreover, CSCs can differentiate into diverse cell types within the tumor, thereby giving rise to the progeny cells that constitute the bulk of the tumor. Notably, key components of the TME, such as CAFs or TAMs, secrete factors that induce EMT. This process contributes to an expansion of CSC subsets, enhances cervical squamous cell carcinoma metastatic potential, and confers resistance against chemotherapy and radiotherapy (55, 56). However, there are challenges in identifying specific markers for cervical squamous cell carcinoma (CCSC), including ABCG2, MSI1, PROM1 (CD133), ITGA6 (CD49f), KRT17 (CK17), SOX2, and Pou5f1 (OCT4). In addition research on CCSCs remains limited.

CSCs can enhance DNA repair capacity via the phosphorylation of ATM and CHK1/CHK2; moreover, they can evade cell death induced by DNA damage by enabling antiapoptotic signaling pathways, such as the Notch, PI3K/Akt, and WnT/β-catenin signaling pathways (57, 58). The C-myc-CHK1/Chk2 signaling axis accommodates the DNA damage checkpoint response, thus leading to CSC resistance against radiotherapy (59); however, the pharmacological inhibition of CHK1 and Chk2 has been demonstrated to enhance the sensitivity of CSCs to radiotherapy and chemotherapy (60). In cervical cancer cells, miRNA125 significantly inhibits the CSC phenotype thereby improving sensitivity to radiotherapy and chemotherapy (61, 62). Additionally, Sonic Hedgehog, PTEN, TFG and other signaling pathways also regulate key activities of CSCs (63); however, their relationship with cervical cancer cells remains unexplored.

## 5 Hypoxia

Ionizing radiation exerts its tumor-killing effects through both direct and indirect mechanisms. The direct effect involves the absorption of radiation in the biological medium, thus leading to direct interactions with target molecules and subsequent cell death. The indirect effect entails the interaction between radiation and nontarget atoms or molecules within cells, thus resulting in the generation of reactive oxygen species (ROS) that ultimately damage target molecules. Hypoxia has emerged as a prominent factor contributing to radiation resistance in tumor cells. Moreover, oxygen depletion diminishes the efficacy of radiation-induced DNA strand breaks (64). Conversely, hypoxic stimulation may enhance cell radiation resistance by modulating cellular signaling pathways and DNA damage repair mechanisms (65).

HIF-1α, which is a significant transcription factor, is upregulated under hypoxic conditions. Radiation enhances the activation of HIF-1, either through the damage to the vasculature or the generation of reactive oxygen species (ROS) (7). Elevated HIF-1α expression preradiotherapy has been proposed as being a predictive biomarker for suboptimal response to preoperative radiotherapy in patients with laryngeal, oropharyngeal, laryngeal, esophageal, and cervical cancer. In terms of metabolism, tumor cells predominantly rely on aerobic glycolysis and elevate glutamine utilization. Additionally, tumor cells exhibit a preference for utilizing glutamine metabolism for replication. Furthermore, tumor cells leverage glutamine metabolism to generate precursors of glutathione and facilitate intracellular antioxidant production. Glutaminase functions as the initiating and rate-limiting enzyme in tumor cell glutamine glycolysis. Glutathione serves as the most significant intracellular antioxidant enzyme, whereby it accelerates oxygen free radical excretion and reduces cellular damage. Recent studies have demonstrated that radiation can stimulate HIF-1α activation and regulate the downstream gene GLS2 to increase its transcriptional activity, thereby promoting heightened glutamine metabolism and augmented production of antioxidant metabolites such as glutathione, NADH, and NADPH; accordingly, cervical cancer cells become resistant to radiation (66). Moreover, under hypoxic conditions, HIF-1 safeguards cervical cancer cells from radiation-induced apoptosis by adjusting the expression of p53 and the vascular endothelial growth factor (65). The overexpression of HOTAIR induces radiation resistance in cervical cancer cells by upregulating HIF-1α expression; however, the mechanism underlying the interaction between HOTAIR and HIF-1α in cervical cancer cells postradiotherapy remains obscure (67).

Interestingly, hypoxia also regulates radiation tolerance by modulating the activity of CSCs. Hypoxia induces a quiescent state in CSCs with reduced proliferation rates to protect them from radiation-induced damage. Conversely, hypoxia activates the PI3K/ATK pathway via HIF1α and HIF2α as a feedback loop, thus promoting the induction and self-renewal of CSCs. Additionally, hypoxia promotes the radioresistant phenotype of ALDH-1 positive CSC-like cells derived from SiHA and HeLa cervical cancer cell lines by facilitating DNA damage repair (68). Finally, hypoxia leads to HIF-1α-dependent upregulation of PD-L1 on TAMs, thus further exacerbating tumor resistance to radiation therapy (46).

## 6 Cell cycle arrest

The cell cycle is categorized into four phases: G1 (the growth phase preceding DNA synthesis), S (DNA replication/synthesis), G2 (final preparations for cell division), and M (mitosis) (69, 70). Upon exposure to ionizing radiation, tumor cells can respond by blocking the cell cycle, thus providing valuable time for self-repair and the avoidance of radiation damage. This results in an increase in resistance to radiotherapy. Checkpoints at the G1/S and G2/M phases play a critical role in governing the cell cycle (71, 72). Normal cells, which are equipped with P53 which is a key regulator of the G1/S checkpoint will stall in the G1 phase following DNA damage and will not advance into the S phase until DNA repair mechanisms are activated. However, tumor cells generally lack functional regulators at the G1/S checkpoint, thus enabling them to readily enter the S phase and often arrest in the G2/M phase (73, 74). It has been noted that the mid-late S phase, early G1 phase, and late G2/M phase are particularly susceptible to radiation (75).

**Figure 4:**
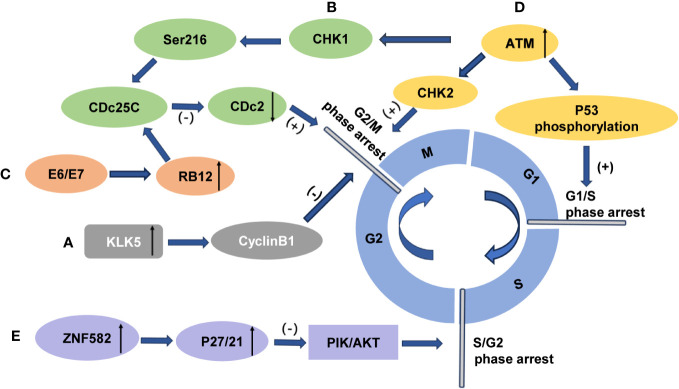
The influence of cervical cancer radiosensitivity by modulation of cell cycle arrest **(A)** Overexpression of KLK5 prevents G2/M phase arrest and enhances the radiosensitivity of cervical cancer cells through the cyclin B1 signaling pathway. **(B)** Overexpression of ZNF582 induces p27/p21 accumulation, inhibits the PI3K/Akt pathway, induces S/G2 phase arrest, and enhances radioresistance in cervical cancer cells. **(C)** Radiation-induced activation of protein kinase CHK1 leads to phosphorylation of protein phosphatase CDc25C at Ser216, which inhibits phosphate removal from Cdc2. Failure to activate CDc2 results in G2/M arrest and enhanced radiation resistance. **(D)** Upregulation of Rab12 by E6 and E7 promotes CDc25C phosphorylation and inhibits CDc2 activation, inducing G2/M arrest and enhancing radiation resistance. **(E)** Activated ATM can induce G2/M phase arrest through ATM-Chk2 and ATR-CHK1 signaling pathways while also inducing G1/M phase arrest through P53 phosphorylation to enhance radiation resistance.

The human tissue kinase family member KLK5 exhibits a broad range of biological functions. Cyclin B1 plays a vital role in regulating mitosis initiation and facilitating the transition from the G2 to M phase (76). It has been demonstrated that an increase in KLK5 expression impedes G2/M phase arrest through cyclin B1, thereby reducing apoptosis and enhancing resistance to radiation therapy (**Figure 4A**). The inhibition of KLK5 in cervical cancer cells decreases radioresistance by downregulating cyclin B1 expression and blocking the transition to the G2/M phase. Consequently, KLK5 can serve as a marker for assessing radiosensitivity and prognosis in cervical cancer patients (75). G2/M phase arrest in HPV-positive cervical carcinoma cells exposed to radiation is mediated by activating the protein kinase CHK1, which can phosphorylate the protein phosphatase CDc25C at Ser216. The dephosphorylation of CDc25C on Thr14/Tyr15 by CDc25C inhibits phosphate removal from Cdc2, thus leading to G2/M arrest when CDc2 fails to activate (77, 78) (**Figure 4B**). The expression of Rab12 has been demonstrated to be significantly upregulated in both cervical cancer tissues and HPV-positive cervical cancer cell lines. Furthermore, HPV oncoproteins–E6 and E7 upregulate the expression of Rab12, thus resulting in radiation resistance. Upon radiation exposure, Rab12 enhances the phosphorylation of CDc25C, thus maintaining CDc2 in an inactive state and exacerbating G2/M phase arrest (75) (**Figure 4C**). Additionally, ATM is thought to contribute to tumor radioresistance by regulating cell cycle arrest. As a serine-threonine protein kinase, ATM controls G2/M phase cell cycle arrest induced by DNA damage following irradiation (79). Upon the occurrence of DNA damage, activated ATM is recruited to phosphorylate multiple substrates to initiate cell cycle arrest as a method to allow sufficient time for DNA damage repair (80, 81). Previous studies have demonstrated a correlation between elevated ATM expression and radiation resistance. It has been suggested that the inhibition of ATM expression can enhance the sensitivity of tumor cells to radiotherapy by preventing ATM-induced activation of ATM-Chk2 and ATR-CHK1 signaling (**Figure 4D**). Furthermore, ATM is involved in regulating the G1/S phase transition. Upon ionizing radiation-induced damage, activated ATM phosphorylates P53 which combines with the negative regulator MDM2, thus leading to cell cycle arrest. Additionally, it facilitates the binding of FBXW7 to P53, thereby regulating P53 degradation and ensuring the restoration of basal levels for proper cancer cell cycle progression while also contributing to radiation resistance (74, 82) (**Figure 4D**). Fu et al. observed an upregulation of ATM expression in residual cervical cancer tumor tissue after radiotherapy. Therefore, increased expression of the ATM gene is believed to play a role in radiation resistance in advanced cervical cancer (83).Sad to say, the underlying mechanism remains unclear.

The intracellular protein transcription factor zinc finger protein 582 (ZNF582) encodes nuclear proteins. It has been suggested that the overexpression of ZNF582 can induce cell cycle arrest at the S/G2 phase in cervical cancer cells treated with radiotherapy. This is achieved through the accumulation of p27/p21, which inhibits the PI3K/Akt pathway and enhances resistance to radiation (84) (**Figure 4E**).

## 7 Epigenetic mechanisms

Epigenetic mechanisms play a crucial role in governing gene expression associated with cell survival and proliferation, the evasion of apoptosis, DNA repair, and EMT regulation. Radiotherapy induces resistance to radiation in cancer cells by reshaping their epigenetic landscape. Currently, the extensively investigated epigenetic mechanisms primarily encompass DNA methylation, histone modification, and noncoding RNA that safeguard cancer cells against the cytotoxic impact of radiation through precise regulation of gene expression (85, 86).

### 7.1 DNA methylation

DNA methylation can be categorized into hypomethylation and hypermethylation. Hypomethylation is generally believed to exert a permissive effect on gene expression. Conversely, hypermethylation exerts a suppressive effect on gene expression (87). The process of DNA methylation primarily involves the action of DNA methyltransferases (DNMTs), which regulate the integrity of the genome and the stability of chromosomes. The upregulation of DNMT in diverse tumors results in hypermethylation and oncogenic stimulation (88, 89). Radiation therapy promotes radiation resistance by modulating DNA methylation to regulate specific transcription factors (90).

In high-risk HPV positive patients with cervical precancerous lesions, the genes GHSR, SST, ZIC1, CADM1, Mal, and miR124 exhibit hypermethylation not only in cervical smears and tissue samples but also show an increasing methylation rate with disease severity. The combination of high-risk HPV positive testing aids in the diagnosis of CIN2+ and the evaluation of prognosis (91, 92). The methylation of EPB41L3 and JAM3 remains unaffected by high-risk HPV and demonstrates good diagnostic accuracy for CIN2+ (93). Furthermore, previous study has indicated that the detection of gene methylation (PHACTR3, PRDM14, GHSR, FAM19A4, SST, and ZIC1) in urine is beneficial for diagnosing cervical cancer (94). One study showed that DNA methylation can inhibit the transcription of miR-138 and promote EMT, proliferation, invasion and metastasis of cervical cancer cells, which is closely related to lymph node metastasis and poor prognosis (95).

**Figure 5:**
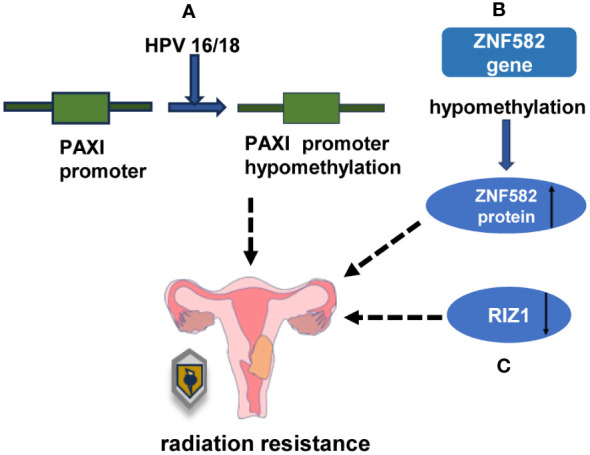
DNA and histone methylation is involved in the regulation of radiation sensitivity in cervical cancer **(A)** HPV16/18 reduced the radiosensitivity of cervical cancer by reducing the methylation of PAX1 promoter in host cells. **(B)** Overexpression of ZNF582 protein could enhance the radioresistance of cervical cancer cells. **(C)** The low expression of RIZ1 decreased the sensitivity of cancer cells to radiation damage.

The hypomethylation of PAX1 is considered to be a risk factor for residual tumor following radiotherapy. It has been suggested that PAX1 methylation is associated with HPV16/18 infection in cervical cancer, which further influences the efficacy of radiotherapy by inducing the methylation of the PAX1 promoter in host cells (96) (**Figure 5A**). Unfortunately, the underlying mechanism behind Pax1-induced radiation resistance has not been reported thus far. In cervical cancer, adenocarcinoma is known to have a worse prognosis compared to squamous cell carcinoma. Wu et al. discovered that hypermethylation of the ZNF582 gene was linked to decreased protein expression, whereas negative ZNF582 gene methylation was associated with higher ZNF582 protein expression. The overexpression of ZNF582 protein reinforced the tolerance of cervical carcinoma cells towards chemotherapy and radiation (97) (**Figure 5B**).In a previous study, it was reported that ZNF582 methylation was detected in 100% of squamous cell carcinoma tissues (97). However, the study by Wu’s observed a low rate of ZNF582 methylation in cervical adenocarcinoma tissues (97).

### 7.2 Histone modification

Histones play a crucial role in nucleosome assembly as central constituents of chromatin, and through the processes of acetylation/deacetylation, methylation/demethylation, and phosphorylation, they shape the epigenetic patterns governing transcriptional processes.

#### 7.2.1 Histone methylation

Histone methylation is regulated by histone methyltransferases (HMTs) and histone demethylases (HDMs), which can ultimately impact cell radiosensitivity by influencing DNA damage repair. Common methylation sites associated with DNA damage repair include H3K4, H3K9, H3K27, H3K36, H3K79, and H4K20. The methylation of H3K4, H3K36, and H3K79 is generally associated with gene activation, whereas the methylation of H3K9, H3K27, and H4K20 is typically linked to gene repression (98). Histone demethylases and methyltransferases have been identified in radioresistant cervical cancer cells. The low expression of RIZ1 (retinoblastoma protein-interacting zinc finger protein), also known as KMT8 (lysine methyltransferase 8), is associated with radiation resistance. The overexpression of RIZ1 enhances the sensitivity of cancer cells to radiation-induced damage (99) (**Figure 5C**).

#### 7.2.2 Histone acetylation

Both DNA and histone proteins are susceptible to methylation, whereas acetylation is exclusively associated with histones (85). Histone acetylation mediated by histone acetylase (HAT) and histone deacetylase (HDAC) alters the spatial conformation of nucleosomes, thereby influencing chromosome structure and regulating gene transcription (100, 101).

**Figure 6:**
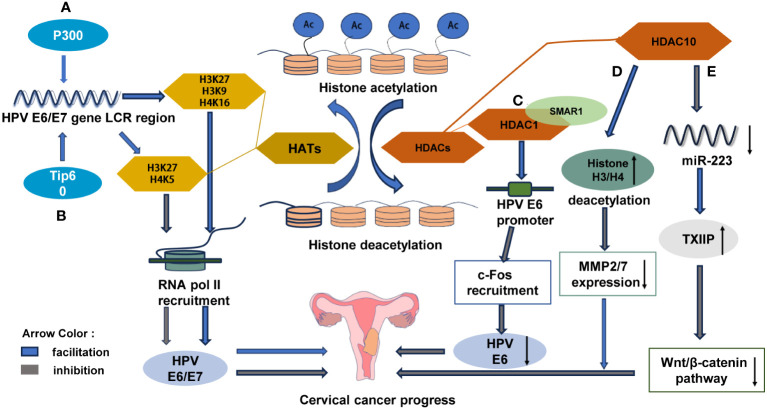
Histone modifications in epigenetic mechanisms are indispensable for the development and progression of cervical cancer **(A)** p300 promotes the acetylation of histone H3K27, H3K9 and H4K16, and enhances the expression of HPVE6/E7 by promoting the recruitment of RNA polymerase (Pol II) to the long control region (LCR) of HPV18 to promote the development of cervical cancer. **(B)** Tip60 inhibits RNA pol II recruitment of HPV18LCR by enhancing H3K27 and H4K5 acetylation, thereby inhibiting HPVE6/E7 expression and suppressing cervical carcinogenesis. **(C)** HDAC1 interacts with the nuclear matrix protein SMAR1 to inhibit the recruitment of c-Fos to the E6 promoter to suppress HPVE6 transcription and suppress cervical cancer development. **(D)** HDAC10 promotes the deacetylation of histone H3 and H4 to suppress the expression of matrix metalloproteinases (MMP2 and MMP7) and further suppress the development of cervical cancer. **(E)** HDAC10 up-regulates TXNIP protein by inhibiting the expression of miR-223, leading to the overexpression of TXNIP inhibiting Wnt/β-catenin pathway carcinogenesis.

Tip60 and p300, as factors of histone acetyltransferase (HAT) activity, play a regulatory role in the genes of human papillomavirus (HPV). In cervical cancer cells, p300 promotes the acetylation of histones H3K27, H3K9, and H4K16, thereby enhancing the expression of the HPVE6/E7 gene by facilitating the recruitment of RNA polymerase II to the long control region (LCR) of HPV18. This ultimately promotes the development of cervical cancer (**Figure 6A**). In contrast, Tip60 enhances acetylation of H3K27 and H4K5 and inhibits the expression of HPV E6/E7, thereby inhibiting the development of cervical cancer by inhibiting the recruitment of RNA pol II of HPV18LCR (102) (**Figure 6B**). HDAC1 interacts with the nuclear matrix protein SMAR1 to inhibit HPVE6 transcription by preventing tumor promoter c-Fos from being recruited. This inhibition leads to the suppression of cervical cancer development (103) (**Figure 6C**). On one hand, HDAC10 inhibits the expression of the matrix metalloproteinases MMP2 and MMP7 by promoting deacetylation at histones H3 and H4, which further hinders the occurrence and progression of cervical cancer (104) (**Figure 6D**). On the other hand, HDAC10 upregulates TXNIP protein expression by suppressing miR-223 expression, which results in the overexpression of TXNIP that inhibits carcinogenesis via Wnt/β-catenin pathway activity thus further restraining CC cell activity (105) (**Figure 6E**). Research has demonstrated that L- and D-lactate inhibit HDAC, thereby promoting hyperacetylation of histone H3 and H4 and activating hydroxycarboxylate receptor 1 (HCAR1) in cervical cancer cells to facilitate DNA repair. This results in the development of resistance in cancer cells against neocarsinotin, doxorubicin, and cisplatin (106). The expression levels of histone deacetylases HDAC4 and HDAC6 showed a negative correlation with overall survival in glioblastoma patients who exhibited poor responses to radiotherapy (107). The subunits CHD4 and CHD3 of the NuRD complex (nucleosome remodeling and deacetylase), which are involved in chromatin remodeling and the activity of histone deacetylase, were found to be higher in radiation-insensitive rectal cancer patients than in radiosensitive patients (108). However, the impact of histone acetylation on the radiosensitivity of cervical cancer remains unexplored in the current scientific literature.

## 8 Noncoding RNA

Noncoding RNAs, including long non-coding RNAs (lncRNAs), microRNAs (miRNAs) and circular RNAs (circRNAs), participate in the regulation of chemoradiotherapy sensitivity in a variety of cancers (109).

### 8.1 LncRNAs

The length of lncRNAs exceeds 200 bp, thus enabling them to exert regulatory functions not only at the transcriptional level but also indirectly enhance the translation of mRNA targets through their role as miRNA sponges in the lncRNA/miRNA/mRNA axis (110).

Wu et al. discovered that lncRNAs have a dual regulatory effect on the radiosensitivity of patients with different malignant tumors. For instance, Gas5 can enhance radiosensitivity, whereas LINC00662, NEAT1, LINP1, MALAT1, SNHG6, HOTAIR and PCAT1 can enhance radioresistance (111, 112).

**Figure 7:**
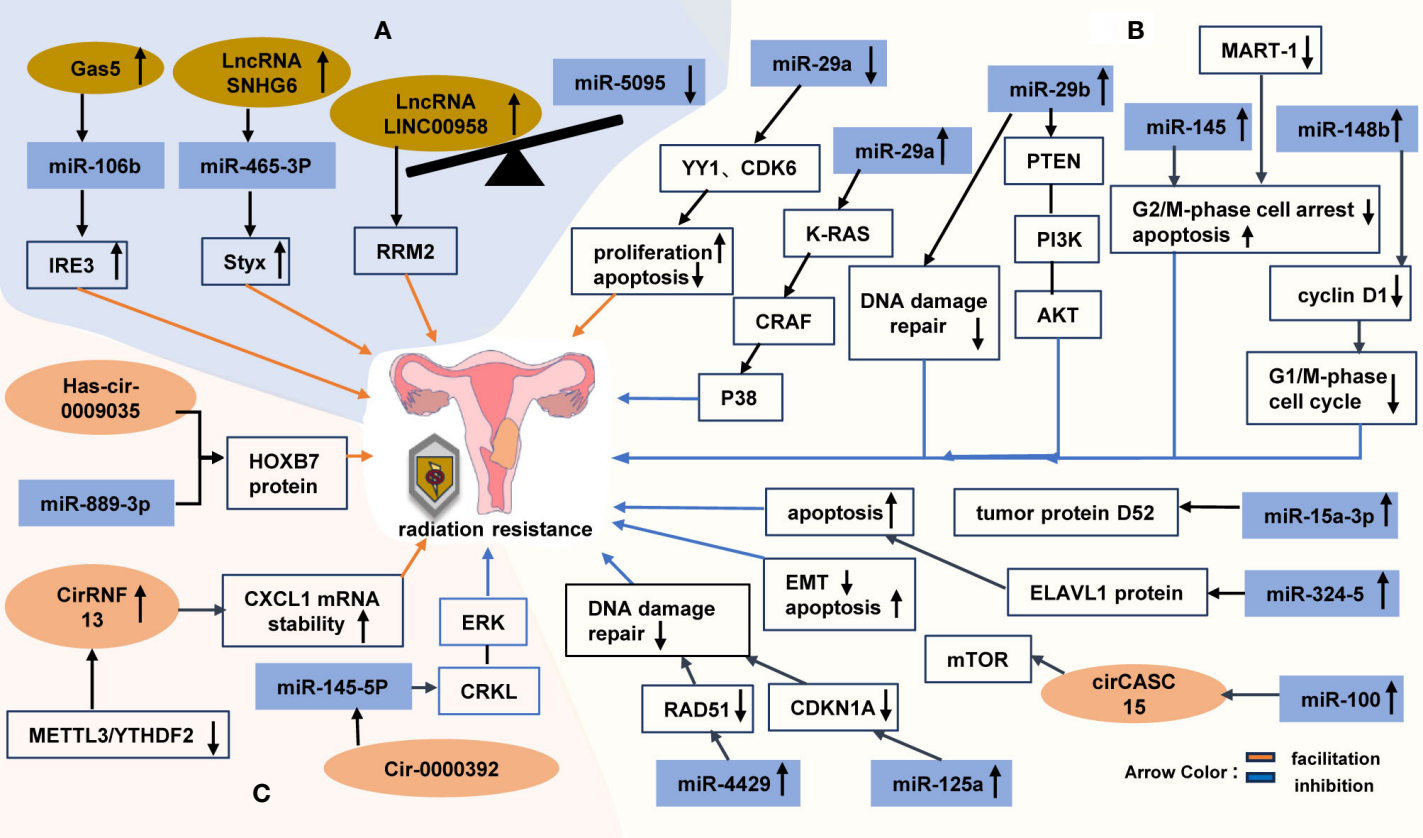
The Non-coding RNAs involved in the regulation of radiation tolerance in cervical cancer **(A)** the relationship between lncRNAs and radiotherapy resistance in cervical cancer. **(B)** miRNAs affect the radiotherapy tolerance of cervical cancer by affecting cell cycle, DNA damage repair and apoptosis through various signaling pathways. **(C)** circRNAs function in radiotherapy tolerance of cervical cancer.

For instance, the overexpression of the tumor suppressor lncRNA growth inhibitor 5 (Gas5) promotes apoptosis in cervical cancer cells by upregulating miR-106b and IER3 to improve sensitivity to radiotherapy and chemotherapy (113, 114). The highly expressed lncRNA SNHG6 in cervical cancer cells promotes cancer cell growth and enhances radiation resistance by regulating the release of Styx from sponge miR-485-3p. However, the knockdown of miR-485-3p or the overexpression of Styx abolishes the effect of silencing of SNHG6 on CC cell growth (115). Zhao et al. found that the highly expressed lncRNA LINC00958 competes with miR5095 to regulate ribonucleotide reductase subunit M2 (RRM2), thus ultimately reducing the sensitivity of CC cells to radiotherapy (116) (**Figure 7A**). Du et al. reported that lncRNA MEG3 increases the sensitivity of CC cells to the chemotherapeutic drug cisplatin through the miR21/PTEN axis (117). Wang et al. discovered that lncRNA UCA1 exerts an inhibitory effect on apoptosis by upregulating cdk2 and downregulating caspase 3, while simultaneously promoting cell proliferation through the upregulation of Survivin levels and the downregulation of p21 levels, thereby enhancing cisplatin resistance in CC cells (118).

### 8.2 MiRNAs

MicroRNAs (miRNAs) are small noncoding RNAs that modulate gene expression by recognizing homologous sequences and exerting regulatory effects on transcription, translation, or epigenetic processes (119). The regulation of miRNAs can activate radiation-related signal transduction pathways, alter cell cycle progression, modulate DNA damage repair pathways, and corresponding mediate the occurrence, development, and sensitivity of tumors to radiotherapy. The promotion effect of miR-182 on distant metastasis of cervical cancer is realized through Wnt/β-catenin axis and its downstream genes (120). Low expression of miR-145 increases tumor invasion and lymphatic metastasis by acting on target genes STAT1, IRS, BNIP3, and C-MYC. The low expression of miR-125b enhances lymph node metastasis of cervical cancer depending on the target gene BAK1 (47).

A previous study demonstrated that the miR-29 family exhibits the highest correlation with miRNA in HPV-infected cervical cancer cells (121). RT leads to a decrease in the expression of the miR-29 family in cervical cancer cells. YY1 and CDK6, which are downstream targets of miR-29a, may contribute to uncontrolled cell proliferation and resistance to apoptotic stimuli in HeLa cells. However, the high expression of miR-29a is believed to enhance radiosensitivity by inhibiting the K-RAS/CRAF/p38 cascade (122). Zhang discovered that overexpression of miR-29b not only suppresses DSB repair but also reduces PTEN/phosphatidylinositol 3-kinase (PI3K)-AKT activity, thereby improving radiosensitivity in cervical cancer cells. Furthermore, the study by Zhang demonstrated that the delivery cancer-specific polyarginine-disulfide bond-linked polyetherimide (PEI) nanocarriers that are externally coated with miR-29b mimics (R11-SSPEI/miR-29b) could enhance radiosensitivity in radioresistant cervical cancer mice (123). MiR-4429 is expressed at low levels in cervical cancer cells (particularly, radioresistant cells), but is highly expressed in radiosensitive cervical cancer cells. By targeting RAD51, which is a key regulator of DNA damage repair, miR-4429 enhances radiosensitivity in cervical cancer cells (124). The elevated expression of miR-125a increases radiosensitivity by specifically downregulating CDKN1A and inhibiting DNA damage repair processes specifically for radiosensitive cervical cancers (125) (**Figure 7B**).

The miR-100 gene is considered to be a tumor suppressor gene, with its expression increased in radioresistant breast cancer cell lines (126), but decreased in cervical cancer. Some studies have suggested that cirCASC15 may act as a sponge for miR-100, thus activating the cell cycle to induce mitosis and reducing EMT through the targeting of miR-100 downstream genes. Therefore, the upregulation of miR-100 can potentially enhance radiosensitivity in cervical cancer cells (127). In HPV-positive cervical cancer patients, miR-145 may be sequestered by Malat-1 sponge. Radiation exposure leads to the overexpression of MALAT-1 and the reduction of miR-145 levels; however, the downregulation of MALAT-1 and the overexpression of miR-145 can sensitize cervical cancer cells to radiation by inducing apoptosis and regulating the G2/M phase (128). By regulating cyclin D1 expression, miR-148b induces cell cycle arrest at the G1/S phase (129). The overexpressing miR-15a-3p enhances radiosensitivity in cervical cancer cells by targeting tumor protein D52 to promote apoptosis (130). Additionally, the targeting of the ELAVL-1 protein via miR-324-5p increases sensitivity to ionizing radiation-induced apoptosis in cervical cancer cells (131) (**Figure 7B**).

### 8.3 CircRNAs

Circular RNAs (circRNAs) are a class of single-stranded noncoding RNAs that assume a circular structure via unconventional splicing or anti-splicing events. Numerous aberrant expression of circRNAs has been observed in various types of cancer, thus suggesting their involvement in the modulation of cancer progression through diverse molecular mechanisms[125]. Intriguingly, recent studies have proposed that additional chemical modifications can either inhibit or enhance the function of circRNAs in multiple cancer types. The dysregulated expression of circRNAs in cancer may result from genetic and/or epigenetic alterations induced by specific mutations affecting key regulators such as spliceosome genes (132). The high expression of hsa_circRNA_101996 and hsa_circ_0067934 can augment the occurrence of lymph node metastasis and reduce the survival rate of patients with cervical cancer by regulating miR-8075, TPX2 and miR-545, EIF3C, respectively (47).

Through high-throughput sequencing and construction of a circRNA-miRNA-target gene interaction network, Yu et al. identified circRNAs associated with radiotherapy resistance in cervical cancer. They validated the four most significantly upregulated circRNAs, including hsa_circ_0004015, hsa_circ_0009035, hg38_circ_0004913, and hsa_circ_0000392, as well as the four downregulated circRNAs: RNA hg38_circ_0013682, hg38_circ_0015954, hsa_circ_0013738, and hsa_circ_0013225 (133). X. Zhao et al. discovered that hSA_CIRC_0009035 regulates the protein expression of the proto-oncogene HOXB7 by targeting miR-889-3p to promote radiotherapy resistance in cervical cancer (134). Tian et al. found that circ_0000392 can enhance radiosensitivity in cervical cancer cells through its regulation of the CRKL/ERK signaling pathway by targeting miR-145-5p (135) (**Figure 7C**). Although it is known to enhance gefitinib resistance in NSCLC HCC827 cells (136), there have been no studies on its role in cervical cancer. None of the remaining five circRNAs have been reported thus far.

The novel m6A-modified circular RNA, CircRNF13, was found to exhibit high expression levels in radioresistant cervical cancer tissues and was observed to enhance radiation resistance by upregulating the stability of CXCL1 mRNA. The degradation of CircRNF13 is regulated by METTL3/YTHDF2, which subsequently influences the stability of CXCL1 and contributes to the enhancement of radiosensitivity in cervical cancer cells (137) (**Figure 7C**). However, there is limited knowledge regarding the impact of circRNAs on cervical cancer radiosensitivity and its underlying mechanism. Thus, further investigations are warranted.

## 9 Conclusions

The standard treatment for locally advanced cervical cancer includes external radiation therapy (EBRT) with chemotherapy followed by intensive brachytherapy. For the past few years, with the deepening of research on cervical cancer radiotherapy, new radiotherapy methods emerge in an endless stream. Immune checkpoint blocking inhibitors (inorganic radiosensitized nanoparticles, for instance AGuIX, and DNA repair inhibitors, for instance Triapine) combined with concurrent chemoradiotherapy can augment the efficacy of chemoradio-mediated cell death, which stimulate the T cell response to produce durable adaptive systemic anti-tumor immunotherapy, and thereby improve the survival hope of patients with recurrent and metastatic cervical cancer (138, 139). Intensity modulated conformal radiation therapy(IMRT)uses smaller beams of different intensity to minimize the dose to critical adjacent structures compared to traditional three-dimensional conformal EBRT. IMRT therapy not only has a high coverage of the planned target area but also reduces toxic gastrointestinal and hematological toxicity and the risk of pelvic fractures (140). Over the years, MRI-guided radiation therapy has received much attention as well. MRI is not only radiation-free compared to CT, but also enables the identification of physiologically radiation-resistant areas based on its strong contrast to soft tissue (141). Therefore, MRI-based IGABT can more accurately locate the tumor boundary to achieve individualized radiotherapy, specifically enhance the radiation of local and pelvic tumors, reduce the radiation dose of neighboring healthy tissues, and thus reduce the occurrence of complications. This has contributed greatly to the survival rate of locally advanced cervical cancer patients (142). Finally, proton therapy is also a research hotspot of cervical cancer radiotherapy in recent years. For proton therapy, which not only can the distal dose of the target area be rapidly decreased to reduce the damage to normal tissues, but most importantly, the damage to the ovary can be reduced to protect the endocrine function of women who need total pelvic EBRT. However, there is still a lack of prospective clinical comparative trials involving protons (140). it is a pity that the complex tolerance of cervical cancer to radiotherapy involves numerous genes, factors, and mechanisms that have yet to be fully elucidated. Nonetheless, as research progresses, novel determinants associated with resistance to radiotherapy in cervical cancer may be identified. Further investigations into these intricate processes could provide valuable insights for the development of innovative therapeutic strategies aimed at mitigating radiation resistance, thus ultimately contributing to extended patient survival, improved quality of life, prevention of tumor progression and recurrence, and the benefit a broader spectrum of cervical cancer patients.

## Author contributions

ML: Writing – review & editing, Writing – original draft. LS: Writing – review & editing, Writing – original draft. YK: Writing – review & editing, Supervision, Funding acquisition. ZW: Writing – review & editing, Supervision, Resources, Funding acquisition.
