# Corrigendum: The research progress on radiation resistance of cervical cancer

**DOI:** 10.3389/fonc.2024.1430862

**Published:** 2024-05-17

**Authors:** Meili Liang, Liying Sheng, Yumin Ke, Zhuna Wu

**Affiliations:** Department of Gynecology and Obstetrics, The Second Affiliated Hospital of Fujian Medical University, Quanzhou, Fujian, China

**Keywords:** cervical cancer, radiation resistance, tumor microenvironment, cancer stem cells, epigenetic mechanisms

In the published article, an author name was incorrectly written as Meli Liang. The correct spelling is Meili Liang.

The authors apologize for this error and state that this does not change the scientific conclusions of the article in any way. The original article has been updated.

